# Effect of Mind Mapping Combined With Behavior Rating Scale on Medical Compliance Behavior of Atrial Fibrillation Patients Underwent Radiofrequency Ablation

**DOI:** 10.1002/brb3.70332

**Published:** 2025-02-11

**Authors:** Huan Xu, Yuncheng Qiu, Yanshan Wang, Kunqing Song, Xuemin Guo, Li Wang, Yonglou Zhang

**Affiliations:** ^1^ Fourth Department of Cardiovascular Medicine Cangzhou Central Hospital Cangzhou Hebei China; ^2^ Operating Room Hebei Cangzhou Hospital of Integrated Traditional Chinese and Western Medicine Cangzhou Hebei China

**Keywords:** atrial fibrillation, medical compliance, mind mapping, radiofrequency ablation

## Abstract

**Background:**

Atrial fibrillation (AF) patients undergoing radiofrequency ablation require diligent postoperative care for optimal outcomes. This study investigated the impact of combining mind mapping with behavior rating scales on medical compliance behavior in this population.

**Methods:**

A total of 108 AF patients post‐radiofrequency ablation were randomly assigned to a control group (*n* = 54) or an intervention group (*n* = 54). Both groups received standard education, while the intervention group additionally received education using mind mapping and behavior rating scales. Compliance was assessed using a medical compliance behavior scale at baseline, discharge, and 1‐ to 3‐month follow‐ups.

**Results:**

The intervention group demonstrated significantly higher medication compliance rates at discharge (86.9% vs. 65.9%, *p* = 0.027), 1 month (73.9% vs. 46.8%, *p* = 0.011), and 3 months (60.9% vs. 36.2%, *p* = 0.023) compared to the control group. They also scored higher in medical compliance behavior across all dimensions and had improved quality of life scores at 3 months post‐discharge.

**Conclusions:**

Integrating mind mapping with behavior rating scales into post‐radiofrequency ablation care significantly enhances medication compliance, medical compliance behavior, and quality of life among AF patients.

## Graphical abstract

In atrial fibrillation patients, integrating mind mapping with behavior rating scales into post‐radiofrequency ablation care significantly enhances medication compliance, medical compliance behavior, and quality of life. The findings highlight the potential of mind mapping as an effective intervention against atrial fibrillation.

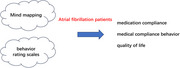

## 1 Introduction

Radiofrequency ablation stands as an innovative approach to addressing atrial fibrillation (AF), a prevalent cardiac condition characterized by irregular heart rhythms (Bosch et al. 2018; Cherian and Callans 2020; Lau et al. 2019; Sagris et al. 2021). This procedure offers a minimally invasive solution to restore normal heart rhythm and alleviate symptoms (Lee and Drake 2023; Mililis et al. 2023). Patient self‐management abilities are pivotal in optimizing the outcomes of radiofrequency ablation (Kolasinski et al. 2020). This encompasses compliance to prescribed medication regimens, embracing heart‐healthy lifestyle modifications, vigilant monitoring of symptoms, attending regular follow‐up appointments, actively seeking education about their condition and treatment options, and accessing emotional support resources as needed (Allegrante et al. 2019; Hester et al. 2018; van Riel et al. 2019). By actively participating in these self‐management practices, patients can play a proactive role in managing their AF and promoting long‐term heart health and well‐being (Baars et al. 2017; Hani and Saleh 2023).

Mind mapping, a visual tool that organizes thoughts and ideas in a structured format, can empower patients to take a proactive role in their recovery journey by helping them outline key aspects of their postoperative care plan, including medication schedules, rehabilitation exercises, dietary guidelines, and symptom monitoring (Palaniappan et al. 2023; Spanoudis and Demetriou 2020). By visually mapping out these elements, patients can gain clarity, set goals, and track progress more effectively, fostering a sense of ownership and empowerment over their health and well‐being (Bai 2023). Additionally, mind mapping can facilitate communication between patients and healthcare providers, allowing for more informed discussions and personalized adjustments to the care plan as needed (Ma et al. 2022; Schaper et al. 2023; Surmeier et al. 2017). Overall, integrating mind mapping into postoperative patient self‐management can enhance patient engagement, compliance with treatment protocols, and ultimately, optimize outcomes and recovery (Russell et al. 2020).

The medical compliance behavior scale serves as a valuable tool in evaluating and enhancing postoperative patient self‐management (Yang et al. 2022). This scale assesses the extent to which patients adhere to prescribed medication regimens, follow lifestyle modifications, attend follow‐up appointments, and engage in other aspects of their care plan (Lavsa et al. 2011; Maria et al. 2023). By quantifying patient compliance, healthcare providers can identify areas of improvement, tailor interventions, and provide targeted support to enhance patient self‐management (Mutneja et al. 2020). Ultimately, the medical compliance behavior scale empowers patients to take an active role in their health by providing feedback and guidance to support their self‐management efforts, leading to improved outcomes and overall well‐being.

Therefore, the purpose of this work is to explore the effect of mind mapping combined with behavioral scales on improving medical compliance behavior in patients undergoing radiofrequency ablation for AF. hope that our study will provide a new theoretical reference for the clinical care of patients after radiofrequency ablation.

## 2 Methods

### 2.1 Participants

**FIGURE 1:**
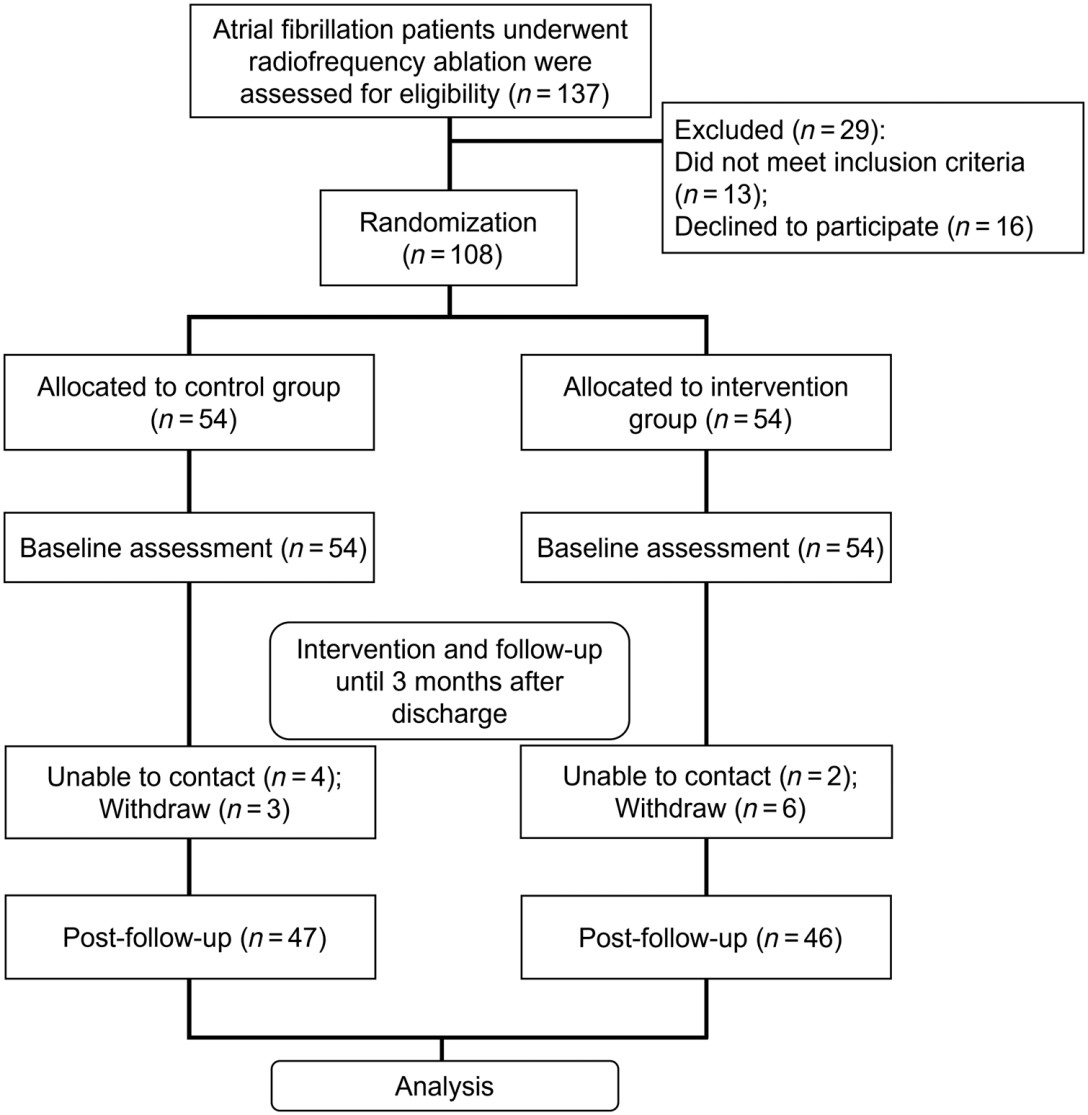
Research framework of this study.

The study enrolled a total of 137 hospitalized patients with AF who underwent radiofrequency ablation in the Cangzhou Central Hospital. The study was approved by Cangzhou Central Hospital, and all the participants signed informed written consent. Thirteen patients did not meet the inclusion criteria, and 16 patients refused to participate, leaving 108 eligible patients who were randomly assigned into either the control group or the intervention group, with 54 patients in each. Both groups received standard health education, with the intervention group additionally receiving health education using mind mapping combined with behavioral assessment scales. Assessments were conducted before intervention, at discharge, and at 1‐ and 3‐month follow‐ups post‐discharge. During the follow‐up period, seven cases in the control group and eight cases in the intervention group were lost to follow‐up. Ultimately, 47 cases in the control group and 46 cases in the intervention group were included in the analysis (Figure 1).

*Inclusion criteria*: Patients eligible for inclusion were those who met the diagnostic criteria outlined in the guidelines for managing AF, had successfully undergone their first radiofrequency ablation procedure for AF, were aged 18 or above, had no mental disorders or consciousness impairments, were able to independently cooperate in completing questionnaire surveys, and voluntarily agreed to participate in the study.

*Exclusion criteria*: Patients were excluded if they had cognitive impairments or were unable to communicate due to reasons such as cultural differences, had physical disabilities or were bedridden for an extended period, or had end‐stage diseases or malignant tumors as comorbidities.

### 2.2 Mind Mapping

Mind mapping involves the expansion of multiple knowledge points from a central keyword, transforming textual information into highly logical connections of lines, vocabulary, and images, facilitating understanding and memory retention. It encompasses disease knowledge as well as guidance on post‐discharge patient observation, self‐pulse measurement, use of handheld electrocardiograms, exercise regimens, dietary recommendations, and emergency techniques for managing complications. The content is comprehensive and richly illustrated. Using MindMaster software, the research team members created mind maps by incorporating selected keywords. The central keyword of the mind map was “atrial fibrillation,” further branching out into seven secondary branches: clinical manifestations of the disease, mechanisms of AF, treatment methods, medication effects and guidance, monitoring indicators (coagulation function, pulse and heart rate, blood glucose, blood pressure), dietary guidance, and activity guidance. Different lines, colors, and images were employed to connect various secondary, tertiary, and quaternary branches. Evaluation was conducted on mind maps created for five eligible research subjects, followed by revisions, culminating in the finalization of the mind map.

### 2.3 Assessment of Patient Compliance Behavior

Patient compliance behavior was evaluated using the Medical Compliance Behavior Scale, which was developed by the researchers based on literature reviews and expert consultations. The scale consists of 78 items across seven dimensions: basic personal information (12 items), accessibility of healthcare services (6 items), influence of medical personnel (9 items), psychological and behavioral factors (24 items), attitudes towards disease (11 items), social support (9 items), and assessment of disease knowledge (7 items). Each item is scored from 0 to 5, with a total score ranging from 0 to 390 points. Higher scores indicate stronger patient compliance behavior. The scale was tested for reliability and validity among post‐radiofrequency ablation patients with AF. Twenty patients participated in an initial questionnaire evaluation, and all found the items clearly described. Based on their feedback, preliminary revisions were made to improve the wording.

### 2.4 Atrial Fibrillation Effect on Quality‐of‐Life

The atrial fibrillation effect on quality‐of‐life (AFEQT) questionnaire evaluates various aspects of quality of life related to AF, including symptoms, treatment worry, impact on daily life, and treatment satisfaction, with higher scores indicating better quality of life.

### 2.5 Preoperative and Procedural Protocol for Ablation

Preoperatively, transesophageal echocardiography should routinely be performed to exclude left atrial thrombus. Anticoagulation therapy should be administered according to the CHA2DS2‐VASc score. If oral anticoagulants are not used, low‐molecular‐weight heparin should be administered subcutaneously for 3 days and discontinued on the day of surgery. All antiarrhythmic drugs (except amiodarone) should be discontinued at least 5 half‐lives prior to the procedure. The patient is positioned supine and connected to electrocardiographic monitoring. After routine disinfection and draping, local anesthesia is administered with a 1% lidocaine injection. Using the Seldinger technique, two punctures are made in the right femoral vein, and two femoral venous sheaths are inserted. A 10‐pole mapping electrode is advanced through one sheath to the coronary sinus. Through the other sheath, a transseptal puncture needle is introduced, and a transseptal puncture is performed. Upon successful puncture, left atrial angiography is conducted, and a PentaRay catheter and an irrigated, temperature‐controlled ablation catheter are advanced to the left atrium. Under CARTO mapping guidance, a 3D model of the left atrium is constructed. Using AI guidance, pulmonary vein isolation is performed with a power setting of 40 W, observing for 15 min to ensure no recurrence of AF. Pulmonary vein electrical isolation is confirmed using a circular mapping catheter. Finally, a standard electrophysiological examination is performed to conclude the procedure.

### 2.6 Statistical Analysis

The analysis was conducted using SPSS 23.0 software. When the measurement data met the assumptions of normality and homogeneity of variance, independent samples *t*‐tests were employed. Nonparametric tests were used when these assumptions were not met. For comparisons involving rates or proportions, the chi‐square test was utilized.

## 3 Results

### 3.1 Demographic and Clinical Characteristics

The demographic and clinical characteristics of patients undergoing radiofrequency ablation for AF were analyzed and are presented in Table 1. The study group comprised 47 patients in the control group and 46 patients in the intervention group. A comparison of baseline data between the two groups revealed no statistically significant differences across various parameters, including age, gender, education level, residence, course of disease, type of AF, EHRA grade, diabetes mellitus, hypertension, coronary heart disease, and stroke. The absence of significant differences in baseline characteristics between the two groups suggests that they were well‐matched, enhancing the comparability of outcomes.

**TABLE 1:** Demographic and clinical characteristics of atrial fibrillation patients received radiofrequency ablation.

Characteristics	Study group		*p*
	Control (*n* = 47)	Intervention (*n* = 46)	
Age			
< 60 years	25 (53.2%)	21 (45.7%)	0.536
≥ 60 years	22 (46.8%)	25 (54.3%)	
Gender			
Male	24 (51.1%)	26 (56.5%)	0.679
Female	23 (48.9%)	20 (43.5%)	
Education level			
High school and below	32 (68.1%)	33 (71.7%)	0.822
College and above	15 (31.9%)	13 (28.3%)	
Residence			
Countryside	21 (44.7%)	18 (39.1%)	0.677
Towns	26 (55.3%)	28 (60.9%)	
Course of disease			
< 3 years	24 (51.1%)	27 (58.7%)	0.534
≥ 3 years	23 (48.9%)	19 (41.3%)	
Type of atrial fibrillation			
Paroxysmal	40 (85.1%)	37 (80.4%)	0.593
Persistent	7 (14.9%)	9 (19.6%)	
EHRA grade			
2a and 2b	37 (78.7%)	32 (69.6%)	0.351
3	10 (21.3%)	14 (30.4%)	
Diabetes mellitus			
Yes	41 (87.2%)	38 (82.6%)	0.575
No	6 (12.8%)	8 (17.4%)	
Hypertension			
Yes	34 (72.3%)	36 (78.3%)	0.632
No	13 (27.7%)	10 (21.7%)	
Coronary heart disease			
Yes	37 (78.7%)	39 (84.8%)	0.802
No	10 (21.3%)	7 (15.2%)	
Stroke			
Yes	43 (91.5%)	40 (86.9%)	0.523
No	4 (8.5%)	6 (13.1%)	

*Note*: Values were expressed as *n* (percentage, %). *p* values were derived from Fisher's exact test.

Abbreviations: EHRA, European Heart Rhythm Association.

### 3.2 Comparisons of Medication Compliance Between the Two Groups at Discharge, 1 and 3 Months After Discharge

Table 2 presents the comparison of medication compliance between the control and intervention groups at discharge, 1 month, and 3 months after discharge. At discharge, significantly more patients in the intervention group demonstrated good compliance compared to the control group (86.9% vs. 65.9%, *p* = 0.027). Similarly, at 1 and 3 months after discharge, a higher proportion of patients in the intervention group exhibited good compliance compared to the control group (73.9% vs. 46.8%, *p* = 0.011 at 1 month; 60.9% vs. 36.2%, *p* = 0.023 at 3 months). Conversely, the proportion of patients with poor compliance was consistently lower in the intervention group compared to the control group across all time points. These findings suggest that the intervention, which incorporated the use of the compliance scale alongside mind mapping in health education, was effective in improving medication compliance among patients undergoing radiofrequency ablation for AF.

**TABLE 2:** Comparisons of medication compliance between the two groups at discharge, 1 month and 3 months after discharge.

Characteristics	Study group		*p*
	Control (*n* = 47)	Intervention (*n* = 46)	
Discharge			
Good compliance	31 (65.9%)	40 (86.9%)	0.027
Poor compliance	16 (34.1%)	6 (13.1%)	
1 Month after discharge			
Good compliance	22 (46.8%)	34 (73.9%)	0.011
Poor compliance	25 (53.2%)	12 (26.1%)	
3 Months after discharge			
Good compliance	17 (36.2%)	28 (60.9%)	0.023
Poor compliance	30 (63.8%)	18 (39.1%)	

*Note*: Values were expressed as *n* (percentage, %). *p* values were derived from Fisher's exact test.

### 3.3 Comparisons of Medical Compliance Behavior Between the Two Groups at Baseline, Discharge, 1 and 3 Months After Discharge

**FIGURE 2:**
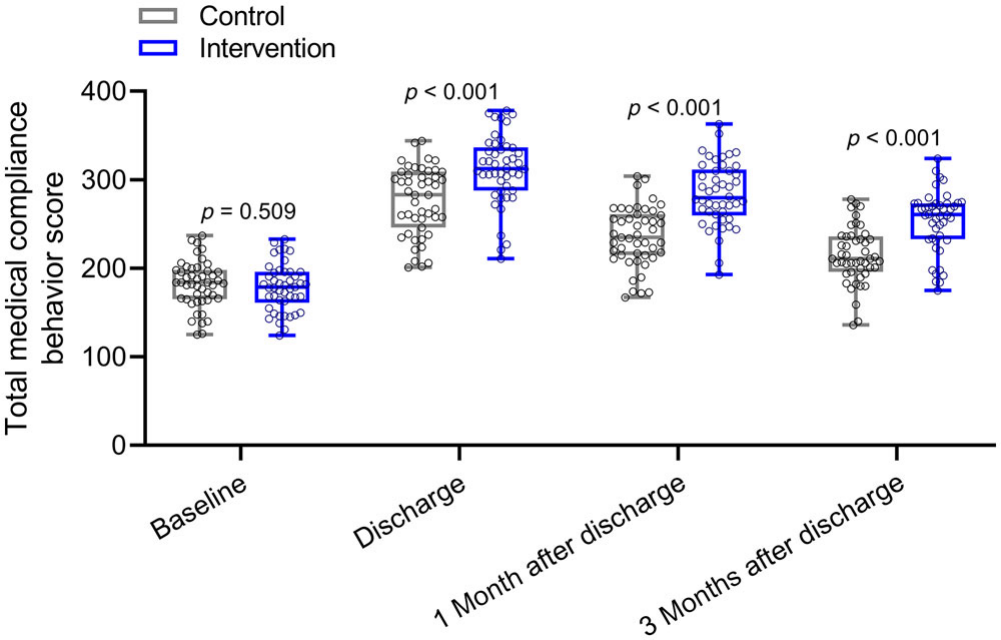
Comparisons of total medical compliance behavior score between the two groups at baseline, discharge, 1 and 3 months after discharge. Data were shown as box plot. *p* values for each group were derived from either unpaired *t*‐test.

Table 3 provides a comprehensive analysis of medical compliance behavior between the control and intervention groups at various time points: baseline, discharge, 1 month, and 3 months after discharge. At baseline, no significant differences were observed between the control and intervention groups across individual dimensions or total scores of the medical compliance behavior scale. However, upon discharge, the intervention group exhibited notably higher scores across all dimensions compared to the control group, indicating superior medical compliance behavior. This trend persisted at 1 and 3 months post‐discharge, with the intervention group consistently demonstrating better medical compliance behavior compared to the control group (Figure 2).

**TABLE 3:** Comparisons of medical compliance behavior between the two groups at baseline, discharge, 1 and 3 months after discharge.

Characteristics	Study group		*p*
	Control (*n* = 47)	Intervention (*n* = 46)	
Baseline			
Basic personal information	23.42 ± 3.16	22.95 ± 3.35	0.315
Feasibility of health services	11.81 ± 1.97	12.06 ± 2.03	0.528
The influence of medical staff	24.29 ± 2.86	24.51 ± 3.11	0.646
Psychological behavior factors	46.82 ± 6.63	45.75 ± 7.75	0.483
Attitude towards disease	30.57 ± 5.24	31.82 ± 5.19	0.278
Social support	28.39 ± 4.95	27.97 ± 5.31	0.388
Disease knowledge survey	16.59 ± 2.74	16.89 ± 2.83	0.427
Total score	181.87 ± 26.34	178.31 ± 26.53	0.509
Discharge			
Basic personal information	43.52 ± 6.11	47.58 ± 6.64	0.012
Feasibility of health services	21.16 ± 2.99	25.87 ± 3.32	0.006
The influence of medical staff	32.47 ± 5.13	37.85 ± 4.48	< 0.001
Psychological behavior factors	80.53 ± 9.28	93.62 ± 10.55	< 0.001
Attitude towards disease	38.41 ± 5.59	46.66 ± 6.73	< 0.001
Social support	32.46 ± 4.98	36.91 ± 5.38	0.008
Disease knowledge survey	25.38 ± 3.44	28.02 ± 3.47	0.016
Total score	276.32 ± 38.52	311.67 ± 39.56	< 0.001
1 Month after discharge			
Basic personal information	36.17 ± 5.08	43.05 ± 6.36	0.003
Feasibility of health services	20.12 ± 3.06	23.23 ± 3.28	0.019
The influence of medical staff	26.34 ± 3.75	31.69 ± 4.11	0.004
Psychological behavior factors	72.56 ± 9.14	85.72 ± 10.49	< 0.001
Attitude towards disease	33.42 ± 4.41	41.73 ± 5.58	< 0.001
Social support	30.17 ± 4.85	33.18 ± 4.99	0.104
Disease knowledge survey	19.01 ± 2.56	25.37 ± 3.16	0.001
Total score	235.92 ± 33.66	284.03 ± 36.15	< 0.001
3 Months after discharge			
Basic personal information	27.45 ± 4.63	40.12 ± 6.63	< 0.001
Feasibility of health services	16.27 ± 2.69	19.44 ± 3.01	0.007
The influence of medical staff	22.13 ± 3.22	27.36 ± 3.79	< 0.001
Psychological behavior factors	68.36 ± 11.06	74.75 ± 11.18	0.009
Attitude towards disease	30.24 ± 4.93	35.58 ± 5.41	0.002
Social support	29.38 ± 4.55	31.32 ± 4.73	0.081
Disease knowledge survey	17.57 ± 3.26	22.05 ± 3.49	< 0.001
Total score	214.38 ± 31.73	252.93 ± 34.63	< 0.001

*Note*: Values were expressed as mean ± SD. *p* values for each group were derived from either unpaired *t*‐test or Mann–Whitney test as appropriate.

Specifically, at discharge, the intervention group showed significantly higher scores in basic personal information, feasibility of health services, influence of medical staff, psychological behavior factors, attitude towards the disease, social support, disease knowledge survey, and total score compared to the control group (*p* < 0.05 for all). Similar results were observed at 1 and 3 months post‐discharge, with the intervention group consistently outperforming the control group in terms of medical compliance behavior across all dimensions and total scores (*p* < 0.05 for all).

These findings underscore the effectiveness of the intervention, which integrated mind mapping and behavioral assessment scales into health education, in promoting and sustaining improved medical compliance behavior among patients undergoing radiofrequency ablation for AF. Statistical significance was determined using either unpaired *t*‐tests or Mann–Whitney tests as appropriate.

### 3.4 Comparisons of Atrial Fibrillation Effect on Quality‐of‐Life (AFEQT) Between the Two Groups at Baseline and 3 Months After Discharge

**FIGURE 3:**
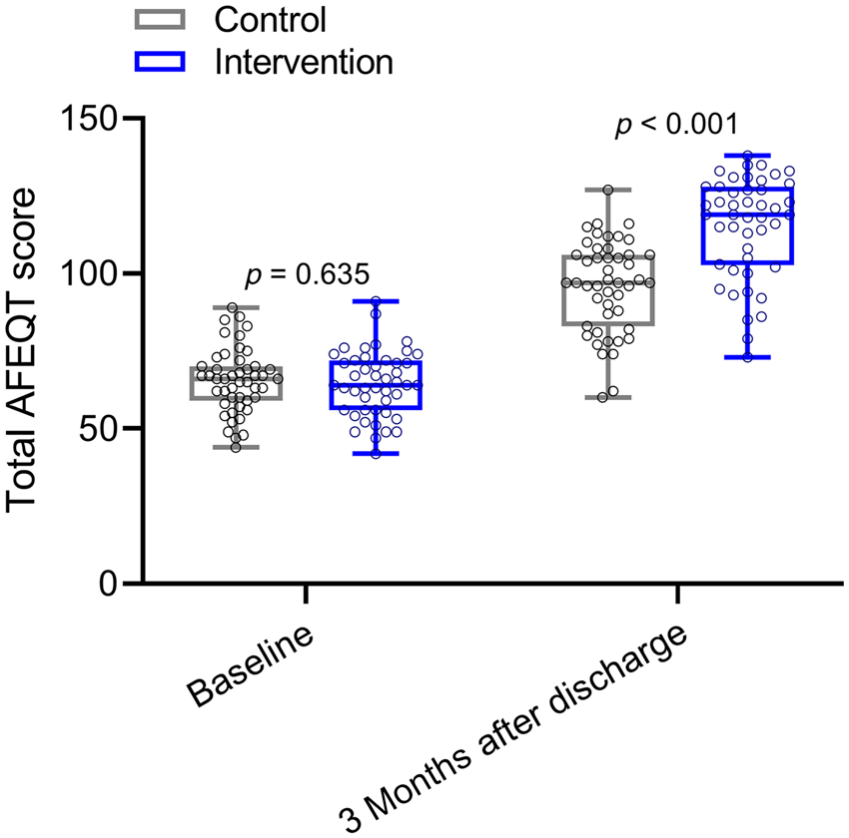
Comparisons of total AFEQT score between the two groups at baseline and 3 months after discharge. Data were shown as box plot. *p* values for each group were derived from either unpaired *t*‐test.

Table 4 presents the comparisons of AFEQT between the control and intervention groups at baseline and 3 months after discharge. At baseline, there were no significant differences between the control and intervention groups in any of the AFEQT dimensions or total score. However, at 3 months after discharge, significant differences were observed between the two groups across all dimensions and the total score. Specifically, the intervention group exhibited higher scores in symptom relief, treatment satisfaction, impact on daily life, and total score compared to the control group (Figure 3). In addition, during the 3‐month follow‐up of the two groups, the recurrence of AF was 11 cases (23.4%) in the 47 cases in the control group and seven cases (15.2%) in the 46 cases in the intervention group. These findings suggest that the intervention, which included mind mapping and behavioral assessment scales in health education, contributed to improvements in quality of life among patients undergoing radiofrequency ablation for AF.

**TABLE 4:** Comparisons of atrial fibrillation effect on quality‐of‐life (AFEQT) between the two groups at baseline and 3 months after discharge.

Characteristics	Study group		*p*
	Control (*n* = 47)	Intervention (*n* = 46)	
Baseline			
Symptom	12.27 ± 2.33	11.96 ± 2.46	0.275
Treatment worry	19.86 ± 3.59	20.14 ± 3.32	0.339
Daily life	26.75 ± 4.41	26.28 ± 4.55	0.536
Treatment satisfaction	6.36 ± 1.15	5.92 ± 1.21	0.218
Total score	65.47± 10.36	64.43 ± 10.54	0.635
3 Months after discharge			
Symptom	20.15 ± 3.16	22.46 ± 2.94	0.014
Treatment worry	27.66 ± 4.78	34.51 ± 5.45	< 0.001
Daily life	40.31 ± 5.83	47.82 ± 6.37	< 0.001
Treatment satisfaction	9.57 ± 1.48	12.11 ± 1.53	0.008
Total score	96.02 ± 14.82	115.17 ± 16.28	< 0.001

*Note*: Values were expressed as mean ± SD. *p* values for each group were derived from either unpaired *t*‐test or Mann–Whitney test as appropriate.

## 4 Discussion

Radiofrequency ablation is the most effective method for curing AF (Baxter et al. 2022). Actively and effectively intervening in factors contributing to recurrence after radiofrequency ablation can significantly reduce recurrence rates and alleviate the burden of the disease (Enriquez and Kattel 2022). Radiofrequency ablation is crucial for the recovery of AF patients. The three‐dimensional radiofrequency ablation technique carried out in our department is at an advanced level domestically, with a success rate of over 80%. For paroxysmal AF, the success rate can exceed 90%. Additionally, this technique is minimally invasive, suitable for the majority of AF patients, and has high patient acceptance (Sinclair et al. 2022). However, postoperative recovery and self‐management are also crucial factors for the success of ablation. Postoperative self‐monitoring of pulse, electrocardiographic monitoring, and detection of asymptomatic recurrent AF play a vital role in the patient's health. Due to limited understanding of the disease and poor medical compliance among most patients, and lacking systematic methods for self‐management, treatment effectiveness cannot be guaranteed. The aim of this study is to explore the effectiveness of mind mapping combined with behavioral assessment scales in improving medical compliance among patients undergoing radiofrequency ablation for AF. Medical compliance refers to the degree to which a patient's behavior aligns with medical advice, directly impacting the efficacy and outcome of the disease. In nursing practice, compliance should be understood from various perspectives, viewing it as a proactive and responsible process where patients closely collaborate with healthcare providers to maintain their health.

This study investigated the impact of a novel intervention combining mind mapping with behavioral rating scales on medical compliance behavior among patients undergoing radiofrequency ablation for AF. The results revealed several noteworthy findings with significant clinical implications. First, the intervention led to significant improvements in medication compliance among patients in the intervention group compared to the control group. Medication compliance is crucial in AF management, as it ensures the optimal efficacy of antiarrhythmic drugs and anticoagulants in maintaining sinus rhythm and preventing thromboembolic events. By enhancing medication compliance, the intervention potentially reduces the risk of AF recurrence and associated complications, thereby improving patient outcomes and reducing healthcare burden.

Second, the intervention positively influenced medical compliance behavior across various dimensions, including basic personal information, feasibility of health services, influence of medical staff, psychological behavior factors, attitude towards the disease, social support, and disease knowledge survey. This comprehensive improvement in medical compliance behavior suggests that the intervention not only addresses medication compliance but also promotes a holistic approach to post‐radiofrequency ablation care. By empowering patients to actively engage in their care plan and adopt healthier behaviors, such as regular follow‐up visits and lifestyle modifications, the intervention may contribute to long‐term disease management and improved clinical outcomes.

Moreover, the intervention resulted in significant enhancements in quality of life among patients in the intervention group compared to the control group at 3 months post‐discharge. Quality of life is a critical outcome measure in AF management, as the condition can profoundly impact patient's physical, emotional, and social well‐being. By addressing patient's psychological well‐being, symptom burden, and treatment satisfaction, the intervention may lead to a more favorable overall experience and improved patient‐reported outcomes.

The clinical significance of this study lies in its potential to transform the standard of care for patients undergoing radiofrequency ablation for AF. By integrating innovative educational strategies like mind mapping with behavioral assessment scales into routine clinical practice, healthcare providers can tailor interventions to individual patient needs, enhance patient engagement, and promote self‐management skills. This patient‐centered approach not only optimizes treatment efficacy but also fosters a collaborative partnership between patients and healthcare providers, ultimately improving patient outcomes and quality of life.

One notable limitation of this study is the relatively small sample size and the potential lack of generalizability of findings to broader patient populations. Conducted in a single center, the study's sample may not fully represent the diverse demographics and clinical characteristics of all AF patients undergoing radiofrequency ablation. Moreover, the loss to follow‐up of a significant proportion of patients introduces the risk of bias and may impact the validity of the results. Additionally, the study did not control for potential confounding variables, such as socio‐economic status or comorbidities, which could influence medical compliance behavior and outcomes. Addressing these limitations in future research through larger, multicenter studies with robust follow‐up protocols and comprehensive adjustment for confounders will be essential to strengthen the validity and applicability of findings.

Future studies should focus on evaluating the impact of mind mapping and behavior rating scale‐based education on patients undergoing ablation with emerging technologies. The continuous evolution of AF ablation techniques, including the use of pulsed field ablation (PFA), balloon‐based systems, and advanced mapping technologies, necessitates tailored educational approaches to optimize patient outcomes. Understanding how these educational tools influence adherence and quality of life in the context of novel technologies will provide valuable insights for clinical practice. Recent key reports on new technologies in AF ablation underscore their potential to transform treatment paradigms. For example, the latest advancements in PFA highlight its safety and efficacy, while novel cryoablation balloon catheters offer promising results in achieving pulmonary vein isolation (Compagnucci et al. 2024; Dello Russo, Compagnucci et al. 2024). Additionally, studies on high‐resolution mapping systems demonstrate significant improvements in arrhythmia detection and ablation precision (Dello Russo, D'Angelo et al. 2024). These developments warrant investigations into how mind mapping and behavior rating scales can enhance patient understanding and compliance in the context of these advanced techniques. By integrating educational innovations with cutting‐edge ablation technologies, future research can identify strategies to improve patient engagement, adherence, and overall clinical outcomes in this rapidly evolving field.

## 5 Conclusions

This study demonstrates that integrating mind mapping with behavior rating scales significantly enhances medical compliance behavior and medication compliance in AF patients undergoing radiofrequency ablation. The intervention group consistently showed better compliance across multiple dimensions, including medication compliance, engagement with healthcare services, and self‐management behaviors, compared to the control group. Furthermore, the intervention led to significant improvements in patient's quality of life, as evidenced by higher AFEQT scores at 3 months post‐discharge. These improvements were observed in areas such as symptom relief, treatment satisfaction, daily life impact, and overall well‐being. The findings underscore the importance of innovative, patient‐centered educational strategies in promoting effective self‐management and fostering a collaborative relationship between patients and healthcare providers. By empowering patients to take an active role in their recovery, this approach not only improves treatment compliance but also enhances the overall quality of life. Future research should explore the long‐term effects of such interventions and assess their scalability and applicability across diverse patient populations.

## Author Contributions

**Huan Xu**: validation, data curation, writing–review and editing, writing–original draft. **Yuncheng Qiu**: validation, writing–review and editing, writing–original draft, funding acquisition, data curation, supervision, resources. **Yanshan Wang**: validation, data curation, writing–review and editing, writing–original draft. **Kunqing Song**: validation, data curation, writing–review and editing, writing–original draft. **Xuemin Guo**: validation, data curation, writing–review and editing, writing–original draft. **Li Wang**: validation, data curation, writing–review and editing, writing–original draft. **Yonglou Zhang**: validation, data curation, writing–review and editing, writing–original draft.

## Ethics Statement

The study was approved by Cangzhou Central Hospital and all the participants signed informed written consent.

## Consent

All participants in this study were informed and gave a written consent. Current study is available from the corresponding author on reasonable request.

## Conflicts of Interest

The authors declare no conflicts of interest.

### Peer Review

The peer review history for this article is available at https://publons.com/publon/10.1002/brb3.70332

## Data Availability

The data could not be shared openly, as required by our department. The raw data could be obtained upon reasonable request to the corresponding author.
